# Continuous Environmental Changes May Enhance Topographic Memory Skills. Evidence From L’Aquila Earthquake-Exposed Survivors

**DOI:** 10.3389/fnhum.2018.00318

**Published:** 2018-08-07

**Authors:** Laura Piccardi, Massimiliano Palmiero, Alessia Bocchi, Anna Maria Giannini, Maddalena Boccia, Francesca Baralla, Pierluigi Cordellieri, Simonetta D’Amico

**Affiliations:** ^1^Department of Life, Health and Environmental Sciences, University of L’Aquila, L’Aquila, Italy; ^2^Neuropsychology Unit, IRCCS Fondazione Santa Lucia, Rome, Italy; ^3^Department of Biotechnological and Applied Clinical Science, University of L’Aquila, L’Aquila, Italy; ^4^Department of Psychology, Sapienza University of Rome, Rome, Italy; ^5^Vincenzo Tiberio Department of Medicine and Health Sciences, University of Molise, Campobasso, Italy

**Keywords:** post-traumatic stress, topographical learning, human navigation, spatial orientation, adaptation mechanisms, earthquakes, natural disasters, trauma-induced sequelae

## Abstract

Exposure to environmental contextual changes, such as those occurring after an earthquake, requires individuals to learn novel routes around their environment, landmarks and spatial layout. In this study, we aimed to uncover whether contextual changes that occurred after the 2009 L’Aquila earthquake affected topographic memory in exposed survivors. We hypothesized that individuals exposed to environmental changes—individuals living in L’Aquila before, during and after the earthquake (hereafter called exposed participants, EPs)—improved their topographic memory skills compared with non-exposed participants (NEPs) who moved to L’Aquila after the earthquake, as only EPs had to modify their previous cognitive map of L’Aquila. We also hypothesized that memory improvement was selective for the navigational space and did not generalize across other spatial and verbal domains. To test these hypotheses, we compared the topographic and spatial memory skills of 56 EPs without post-traumatic stress disorder (PTSD) symptoms to the skills of 47 NEPs using the Walking Corsi Test (WalCT; memory test in the navigational space) and the Corsi Block-Tapping Test (CBT; visuospatial memory test in the reaching space); EPs and NEPs were matched for gender, education and general navigational skills. A sub-group of participants also underwent the Rey-Auditory Verbal Learning Test (RAVLT; verbal memory test). The results showed that only EPs had better performances on topographic learning (TL) assessed using the WalCT rather than spatial learning assessed by the CBT. This outcome suggests the possibility that EPs specifically improved topographic memory. This effect may be due to continuous exposure to environmental changes that have required individuals to learn novel paths within the city and integrate novel information, such as “new towns,” into their pre-existing mental representation of the city. Implications and limitations of the study are discussed.

## Introduction

**Figure 1:**
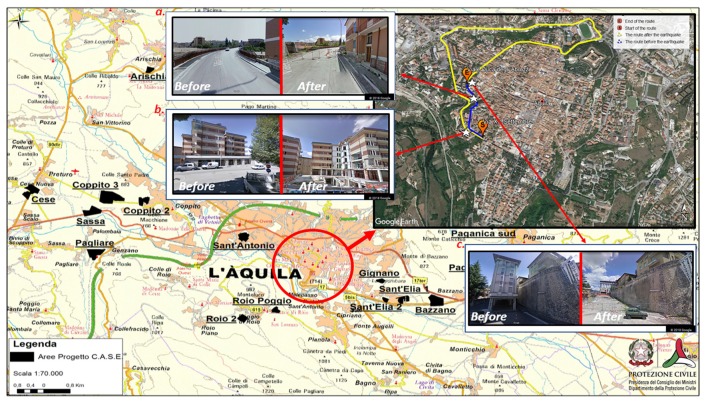
City map of L’Aquila before and after the earthquake. The figure shows the deep, urban modifications that inevitably forced citizens to re-learn the paths of their city after the earthquake. The background map shows: 1) the city center (the red circle; 2) suburbs, where the “new towns” (depicted in black on the map) were built for citizens whose houses were destroyed during the earthquake. The map was derived from the website of the Italian Civil Protection (decree n. 6, 11 May 2009. Source: http://www.protezionecivile.gov.it/jcms/it/view_dossier.wp;jsessionid=162AA553223F304BCBBADDF517785517?contentId=DOS282). On the top right panel of the figure, the map focuses on the changes of L’Aquila city center. Here, a typical route between a starting (S) and an ending (E) point is depicted as it appeared before (blue line) and after (yellow line) the earthquake. The path from S to E was quite short before the earthquake (approximately 0.6 km). However, due to the presence of blocked routes, going from S to E after the earthquake required a very long path (approximately 3.4 km). The map was created using Google Earth^©^ 2018 and Google Maps^©^ 2018. After the earthquake, landmarks along the route dramatically changed. Examples are provided in the a–c boxes (a the bridge “ponte Bel Vedere”; b the students’ dormitory; c the “Duca degli Abruzzi” hotel). The three white stars indicate their positions on the map.

The L’Aquila earthquake produced important environmental changes, which encompassed more than 45 towns and small villages. One year after the earthquake, the city center resembled a “ghost town” (Alexander, 2010, 2013; Díez, 2012; Contreras et al., 2014), with some areas remaining off-limits to citizens. Reconstruction and urban changes are still taking place almost 10 years later. On the one hand, isolated reconstruction initiatives have focused on individual buildings, without a holistic plan for urban recovery (Contreras et al., 2014); on the other hand, a large number of initiatives have included building new houses in settlements outside of the city center. This has forced individuals living in L’Aquila before, during and after the earthquake to acquire new spatial knowledge about the new districts (see Figure 1). The characteristics of such an urban plan pose important questions related to the spatial navigational skills of exposed survivors.

Boccia et al. (2016) found that post-traumatic stress disorder [PTSD: a psychological consequence of a traumatic event involving alterations in behavioral, psychological, physiological, biological and social responses (American Psychiatric Association, 2013)] has both a common network, which spans from the parietal to the frontal cortex and includes limbic structures, and specific networks that are more related to the type of stressor (e.g., the parahippocampal gyrus, the superior temporal and frontal gyri and the middle frontal gyrus, for natural disasters). Some of these structures play key roles in human navigation given that, in the presence of natural disasters, it is possible to observe functional changes in the neural networks since they relate to the perception of surrounding environments and familiar places that have been disrupted by the disaster; for example, the parahippocampal gyrus is involved in scene/place perception and environmental spatial navigation (Epstein and Morgan, 2012; Boccia et al., 2014). Meanwhile, Piccardi et al. (2016a) found that PTSD due to natural disasters modified the cerebral network, e.g., the insula, the lingual gyrus and the inferior and superior frontal gyri in the right hemisphere, involved in learning spatial sequences in the environmental space. These cerebral areas are related to different spatial abilities: the lingual gyrus and insula are involved in learning sequences in the navigational space with specific and complementary contributions (Nemmi et al., 2013). The inferior frontal gyrus is activated during the mental rotation of 3-D objects and letters (Jordan et al., 2001), while the superior frontal gyrus is involved in maintaining spatial orientation in working memory (Cornette et al., 2001).

Interestingly, Tempesta et al. (2012) found that individuals with PTSD caused by the L’Aquila earthquake showed a deficit in forming a cognitive map of a virtual environment although they had spared skills in using the map. The authors interpreted their results as a consequence of hippocampal alterations that have also been reported in patients with PTSD (e.g., Bremner et al., 1999; Bremner, 2001; Shin et al., 2004). Accordingly, Iaria et al. (2007) showed activation of the bilateral hippocampi during the formation and use of a cognitive map (Iaria et al., 2008). Finally, the results by Tempesta et al. (2012) are also consistent with sleep disturbances that participants in their study experienced, which may have led to impaired sleep-dependent spatial memory consolidation.

What about exposed-survivors who do not develop PTSD? In spite of the impact natural disasters have on communities, a large number of exposed survivors undergo a natural recovery process without experiencing any psychopathological consequences (Boscarino et al., 2005; Bonanno et al., 2010). According to the stress-habituation model (Meichenbaum and Novaco, 1985; Jaycox et al., 1998), the effects of a traumatic stressor decrease over time as people adapt to the stress exposure. Although chronic exposure to stress can lead to negative outcomes such as exhaustion, cognitive dysfunction, avoidance behavior and depression (Juster et al., 2010), low or moderate levels of acute stress can be adaptive. For example, it has been found to increase social behaviors such as mutual contact and searching for reassurance from others (Schuster et al., 2001). Traumatic events may also result in different physical and behavioral outcomes, as well as differences in the probability of developing PTSD, as a consequence of individual factors such as personality, gender, age and genetic factors (Ditlevsen and Elklit, 2012; D’Amico et al., 2013; Santiago et al., 2013; Husarewycz et al., 2014; Perrin et al., 2014; Giannini et al., 2016; Piccardi et al., 2016b). Thus, adaptation is crucial to survival, and individual differences in cognitive and emotional responses to both the stressor and context have been found to be key factors in determining outcomes, e.g., anticipation, appraisal, coping, learning and other types of information processing (Hobfoll, 1989; Lazarus, 1991; Holahan et al., 2000; Ironson et al., 2000). An allostatic response, that is, maintaining stability through change, along with adaptation to a current stressor may be considered as a two-stage process that includes central and peripheral allostatic accommodation. Allostatic accommodation encompasses not only the state of being in “homeostatic imbalance” (Sapolsky et al., 2000) but also the process of either bringing the system back to its original equilibrium or finding a new one (adaptive plasticity). The evidence stemming from the mechanisms underlying the post-traumatic growth (PTG)—namely positive psychological changes such as personal resilience, resetting life priorities and openness to new possibilities resulting from major life crises or traumatic events—seems to suggest that in the absence of PTSD, trauma exposure can lead to different responses to the stressor.

Environmental changes occurring after natural disasters such as earthquakes may foster individual skills as they force individuals to re-learn environmental information and acquire new spatial knowledge. Accordingly, spatial ability is understood to be widely affected by experience, for instance, playing video games (Dorval and Pépin, 1986; De Lisi and Wolford, 2002; Feng et al., 2007), orienteering (González et al., 2013; Schmidt et al., 2016), geo-caching (Barnikel et al., 2014; Ellbrunner et al., 2014) and other experiences or targeted training procedures (Cavallini et al., 2003; Boccia et al., 2017) seem to influence spatial ability in everyday life. De Lisi and Wolford (2002) found that girls improved two-dimensional mental rotations and performed at the same level as boys through practice with the popular videogame Tetris. Other studies have found that playing video games may also result in an improvement of topographic orientation in daily life (e.g., Kass et al., 1998; Rafi et al., 2005).

Until now studies have focused on spatial orientation and visuospatial memory skills in individuals suffering from PTSD, almost neglecting the possible modifications in spatial orientation skills in individuals exposed to traumatic events but who never showed signs of PTSD. Here, we aimed to fill this gap by focusing our investigation on topographic and visuospatial memory skills in young individuals who were exposed to the 2009 L’Aquila earthquake but never developed PTSD symptoms. To this end, individuals who were exposed to the L’Aquila 2009 earthquake (exposed participants, EPs) and individuals who were not exposed to the earthquake (non-exposed participants, NEPs) and moved to the city after the disaster were enrolled in the study. Our hypothesis is as follows: EPs had been exposed to continuous environmental changes soon after the earthquake and in the years following it. Due to the large number of modifications occurring in L’Aquila after the earthquake, EPs may have modified their topographic learning (TL) skills as happens for individuals exposed to other experiences such as geo-caching, orienteering and navigational training. In this light, environmental changes that occurred after the earthquake should have acted as navigational training. It is also possible that an optimal level of stress may have improved general memory skills, as predicted by PTG. In the first case, we would expect to see a specific improvement in TL skills compared with visuospatial and verbal memory skills. Otherwise, we would observe general memory improvement across different verbal and spatial domains.

## Participants

A total of 103 college students participated in this study (37 males and 66 females; mean age = 24.48 ± 3.10 years; age range = 19–35 years). They were recruited from the Department of Life, Health and Environmental Science of the University of L’Aquila. Participants were enrolled on a voluntary basis from January 2016 to June 2018. The sample was divided in two groups according to their exposure to the April 6, 2009 earthquake that occurred in L’Aquila, Italy: 56 EPs, including 34 females and 22 males (mean age = 24.9 ± 3.52 years; SE = 0.47; range = 19–35 years), and 47 NEPs including 32 females and 15 males (mean age = 24 ± 2.46 years; SE = 0.36; range = 19–33). The EPs were either in the metropolitan area or a nearby district on April 6, 2009 (from 1.65 km to 20 km from the epicenter of the earthquake). They lived in or nearby L’Aquila before and after the earthquake. The NEPs had never experienced an earthquake, had never been to L’Aquila before the earthquake, and had started to attend the University of L’Aquila only 2–3 years after the earthquake. An initial interview indicated that none of the participants had neurological or psychiatric disorders or alcohol/drug addictions.

To evaluate the presence and nature of the traumatic events on the participants in the 6 months before testing, a trauma symptom inventory (TSI) was obtained (Briere, 1995; Italian Version: Gambetti et al., 2011); none of the participants showed PTSD symptoms. The EPs and NEPs were also compared on each clinical scale of the TSI in order to exclude any possible confounding effects. The two groups were matched for anxious arousal (*t*_(101)_ = 0.06, *p* = 0.95), depression (*t*_(101)_ = 0.88, *p* = 0.38), anger or irritability (*t*_(101)_ = −0.75, *p* = 0.45), intrusive experience (*t*_(101)_ = 0.42, *p* = 0.67), defensive avoidance (*t*_(101)_ = 1.46, *p* = 0.15), dissociation (*t*_(101)_ = 0.94, *p* = 0.35), sexual concerns (*t*_(101)_ = −0.92, *p* = 0.36), impaired self-reference (*t*_(101)_ = −0.20, *p* = 0.84), dysfunctional sexual behavior (*t*_(101)_ = −2.39, *p* = 0.019) and tension-reduction behavior (*t*_(101)_ = −2.39, *p* = 0.019). A significant threshold was set at *p* = 0.05/10 = 0.005 by using Bonferroni’s correction for multiple comparisons.

The participants’ sense of direction and familiarity were evaluated using the “Familiarity and Spatial Cognitive Style Scale” (FSCS; Piccardi et al., 2011b; Italian version: Nori and Piccardi, 2012). None of the participants reported navigational deficits or developmental topographic disorientation (Iaria et al., 2005, 2009; Bianchini et al., 2010). The two groups did not differ in their abilities to read a schematic map or to follow the path indicated on a map (*t*_(101)_ = −1.04, *p* = 0.3) as demonstrated by scores on a screening test (Semmes Test; Semmes et al., 1955).

A sub-group of participants composed of 40 EPs (24 females and 16 males, mean age = 25 ± 3.34, SE = 0.5) and 32 NEPs (24 females and 8 males, mean age = 24 ± 2.33, SE = 0.41) were also asked to perform a verbal memory test to check verbal memory functioning.

All participants signed a written consent form. The experiment was conducted in accordance with the ethical principles for human experimentation outlined in the Declaration of Helsinki. The study was approved by the Institutional Review Board of L’Aquila University.

## Materials and Methods

The following tests were administered to all participants:

### Walking Corsi Test (WalCT: Piccardi et al., 2008b, 2013)

**Figure 2:**
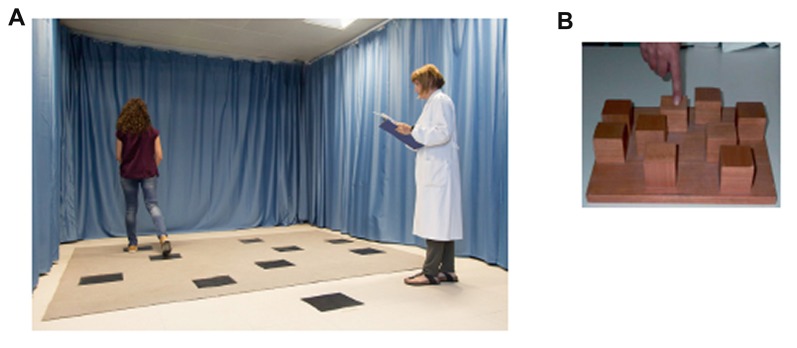
**(A)** The Walking Corsi Test (WalCT): examiner and participant while performing the task. Written informed consent was obtained from the subjects represented in the figure for publication of this experiment. A copy of the written consent is available for review by the Editor-in-Chief of this journal. **(B)** The Corsi Block-Tapping Test (CBT) apparatus.

Walking Corsi Test (WalCT) is a large-scale version of the Corsi Block Tapping Test (CBT; Corsi, 1972) and has been repeatedly used in experimental and clinical practice (e.g., Piccardi et al., 2008b, 2010, 2015; Bianchini et al., 2010, 2014a,b; Nemmi et al., 2013; Palermo et al., 2014; Verde et al., 2015; Palmiero et al., 2016; Tedesco et al., 2017) to assess memory of short paths in a vista space. According Wolbers and Wiener (2014), the vista space is “the space that can be visually apprehended from a single location or with only little exploratory movements…. Typical examples for vista spaces are single rooms or town squares such as the St. Peters Square in Rome” p. 3. Nine black squares (30 × 30 cm) were placed on a floor within a layout, together with a starting point located outside the layout (see Figure 2A). Here, two aspects of topographic long-term memory were assessed, namely, TL and topographic delayed recall (TDR). The examiner showed a fixed 8-square sequence by walking and stopping on each square. The participant was instructed to reproduce the same sequence after the examiner has presented it. The learning criterion, indicating that learning was achieved, corresponded to three consecutive correct reproductions without additional demonstrations by the examiner. If the participant did not achieve the learning criterion, the sequence was repeated by the examiner for a maximum of 18 trials. No feedback regarding performance was provided. During each trial, the number of correct squares reproduced was registered and used for the final score. The learning score was computed by adding the number of correct squares on each of the 18 trials (maximum score: 144). After 5 min, the examiner asked participants to reproduce the 8-square sequence again in a single attempt. The number of squares correctly reproduced was computed and used as a measure of TDR (maximum score: 8).

### Visuospatial and Verbal Memory

The following tests were used to control for the specificity of the TL effect and to exclude general memory impairment in the sample.

#### Corsi Block-Tapping Test (CBT: Corsi, 1972; Italian Version: Piccardi et al., 2013)

The CBT consists of nine blocks (4.5 × 4.5 cm) fixed on a baseboard (30 × 25 cm) in a scattered array (Figure 2B). Two aspects of visuospatial long-term memory in the reaching space were tested: visuo-spatial learning (VSL) and visuo-spatial delayed recall (VSDR). In the VSL, the participants had to learn an eight-block sequence presented by the examiner. The experimenter tapped the series of eight blocks at a rate of one block every 2 s, after which the participant had to tap the same block sequence in the same order it was presented. The learning criterion was reached if the participant reproduced the correct sequence three times in a row (max number of trials: 18). The learning score was calculated by attributing one point for each block correctly tapped until the criterion was reached. This was then added to the score corresponding to correct performance of the remaining trials (up to the 18th; maximum score: 144). Five minutes after the VSL was completed, the VSDR was administered. The examiner asked participants to reproduce the previously learned 8-block sequence. Scores were calculated based on the number of blocks correctly reproduced (maximum score: 8).

#### Rey’s Auditory Verbal Learning Test (RAVLT: Rey, 1958; Italian Version: Carlesimo et al., 1996)

Two aspects of verbal memory were tested: verbal learning (VL) and verbal delayed recall (VDR). The examiner read aloud a list of 15 words at the rate of one per second. The participants were then asked to repeat all of the words from the list that they could remember. This procedure was carried out a total of five times (maximum score: 75). After a 15-min delay, the participants were asked to recall as many words as possible from the first list (maximum score: 15).

All participants were tested individually in a quiet laboratory room with artificial lighting and seated facing the examiner on a height-adjustable office chair during the article and pencil tests (i.e., CBT, RAVLT, FSCS and TSI). They were brought to an adjacent experimental room, without landmarks, where the WalCT and Semmes Test were located.

The test administration was randomized for each participant in order to avoid possible mental fatigue and learning facilitation effect during the experiment.

### Statistical Analysis

All scores were transformed into percentages in order to make them comparable. Two mixed factorial ANOVAs with Group (EPs vs. NEPs) and Apparatus (CBT vs. WalCT) as the independent variables were performed for the learning and delayed recall scores. The groups’ performances on the VL tasks were also compared using ANOVA 2 × 2.

A Bonferroni correction was applied using a significance threshold of *p* = 0.05/2 = 0.025, after correcting the *p*-level for two ANOVAs.

## Results

Descriptive statistics for each variable are shown in Table 1.

**Table 1:** Means, standard deviations and standard errors for each variable of interest in both groups.

	Exposed participants			Non-exposed participants		
	Mean	S.D.	S.E.	Mean	S.D.	S.E.
WalCT-TL	134	8.13	1.7	130.4	16.6	1.85
WalCT-TDR	7.7	1.04	0.14	7.74	0.9	0.13
CBT-VSL	129	15.5	1.9	132.4	12.3	2.06
CBT-VSDR	7.11	1.88	0.25	7.32	1.66	0.24
Rey-VL (*N* = 72)	51.9	9.76	1.54	51.97	9.58	1.7
Rey-VDR (*N* = 72)	11.45	3.27	0.52	12.28	2.27	0.40

*Raw scores for each variable of interest; raw scores were transformed into % values to compare participants’ performance on the different tests. Legend: S.D., Standard Deviation; S.E., Standard Error; WalCT-TL, Walking Corsi Test—Topographic Learning; WalCT-TDR, Walking Corsi Test—Topographic Delayed Recall; CBT-VL, Corsi Block-Tapping Test—Visuo-Spatial Learning; CBT-VDR, Corsi Block-Tapping Test—Visuo-Spatial Delayed Recall; Rey-VL, Rey’s Auditory Verbal Learning Test—Verbal Learning; Rey-VDR, Rey’s Auditory Verbal Learning Test—Verbal Delayed Recall*.

### Results on Topographic Learning and Delayed Recall

The mixed factorial ANOVA for learning scores (TL vs. VSL) revealed a significant group-by-task interaction (*F*_(1,101)_ = 6.693, *p* = 0.011; partial *η*^2^ = 0.062). *Post hoc* pairwise comparisons, performed using Bonferroni’s procedure, showed that EPs were better able to learn a navigational path (WalCT-mean = 134–93%; SE = 1.7) than the visuospatial sequence (CBT–mean = 129–89.5%; SE = 1.9; *p* = 0.013). This result was also replicated in the sub-group of participants: (*F*_(1,70)_ = 6.488, *p* = 0.011; partial *η*^2^ = 0.06; Bonferroni’s correction: *p* = 0.013). The main effects of Group (*F*_(1,101)_ = 0.004, *p* = 0.95) and Task (*F*_(1,101)_ = 1.33, *p* = 0.25) were not significant.

The mixed factorial ANOVA on delayed recall (TDR vs. VSDR) revealed a significant main effect for Task (*F*_(1,101)_ = 6.63, *p* = 0.011; partial *η*^2^ = 0.011). The delayed recall of the topographic task (WalCT—mean = 7.72–96.5%; SE = 0.1) was higher than the delayed recall of the visuospatial task (CBT—mean = 7.21–90.2%; SE = 1.8). This result was not replicated in the sub-group of participants: (*F*_(1,70)_ = 1.76, *p* = 0.19). The main effect of Group (*F*_(1,101)_ = 0.401, *p* = 0.53) and the group-by-task interaction (*F*_(1,101)_ = 0.173, *p* = 0.68) were not significant.

### Verbal Memory Learning and Recall

In the sub-group of participants performing VL and VDR, the comparison between EPs and NEPs (two different ANOVAs were performed) revealed no significant effects (VL: *F*_(1,70)_ = 0.0009, *p* = 0.98; VDR:*F*_(1,70)_ = 1.492, *p* = 0.23).

## Discussion

Topographic memory is crucial for environmental spatial navigation. It is also used for processing and storing information about the environment, such as landmark features and specific locations, as well as spatial relations between landmarks (Berthoz, 1997; Kessels et al., 2001; Piccardi et al., 2008b; Palmiero and Piccardi, 2017). It differs from other forms of memory (i.e., verbal and visuo-spatial) as suggested by evidence from normal developmental processes (Piccardi et al., 2014a,b), neuroimaging data (Nemmi et al., 2013) and classical interference paradigms (Piccardi et al., 2015; Verde et al., 2016). In patients with brain injuries (Piccardi et al., 2008a, 2011a), people with drug-resistant epilepsy (Piccardi et al., 2010) and healthy individuals affected by developmental topographic disorientation (e.g., Bianchini et al., 2010), it is possible to observe selective impairments in topographic memory but not in visuospatial memory for reaching spaces (e.g., Piccardi et al., 2010, 2011a). Because of this, tests that require moving towards and reaching locations, along with those that require remembering paths in real or virtual environments, are needed to detect the presence of impairments in topographic memory. In this study, participants were asked to learn, memorize and recall new paths (square sequences) in the WalCT with the expectation that individuals who were exposed to the L’Aquila earthquake would perform better than individuals who arrived and lived in L’Aquila in the years following the earthquake stemmed from the challenges confronted by the former group after having to continuously re-learn city paths (see Figure 1 that maps environmental changes that happened in L’Aquila after the earthquake).

In some ways, the EPs group had been exposed to navigational training as their city map had been continuously updated over time.

After investigating the topographic memory skills in our sample, we found that EPs obtained higher scores and needed fewer repetitions to learn a new path in the navigational space than in the reaching space. This demonstrates a selective improvement of topographic memory with respect to visuospatial memory. Once a path was learnt however, it was delay-recalled equally well by both groups and, within the EPs group, there was no difference between information-delayed recall in the reaching and in navigational spaces. This result suggests that being exposed to environmental changes after a natural disaster may foster the acquisition of new topographic knowledge but not its recall.

The present results deserve consideration, especially when considered together with the findings by Tempesta et al. (2012) who observed a deficit in forming cognitive maps of the environment in PTSD individuals assessed 1 year after the earthquake. Differences between the present results and those by Tempesta et al. (2012) may be due to the extensive time (about 9 years) that had passed since the earthquake. The absence of differences between the EPs and NEPs groups in our study 9 years after the earthquake does not imply that there were no differences in the EPs closer to the adverse event. Instead, the differences may be due to the presence of PTSD in participants from the previous study. In our study, possible resilience mechanisms in individuals who never developed PTSD may have fostered memory skills. This is consistent with the PTG prediction. However, a general PTG effect should be detected in spatial, topographic and verbal memory. Finding that the EPs showed better topographic, rather than visuospatial, memory point to the hypothesis that navigational changes acted as training in the EPs. Additionally, finding dissimilarities among different types of memory tested—namely, between the WalCT and CBT—observed 9 years after the event suggests that topographic memory, contrary to other forms of memories, could have been continuously trained from the time of the earthquake.

These data are in line with remarks by Edelman (1987) who pointed out that memory could be considered an adaptive and dynamic capacity that implies context-dependent reactivation and one that provides a re-categorization of past information based on the present. As Schachtel (1947) declared, memory is the “capacity for the organization and reconstruction of past experiences and impressions in the service of present needs, fears and interests.”

It is worth noting that this advantage specifically concerns topographic memory rather than visuospatial memory and is not generalizable to other navigational skills, neither as self-referred (as supported by FSCS interview) nor as tested with the Semmes Test. The enhancement of topographic memory appears to be specific and not generalizable to other forms of memory. These results are in line with the effects of navigational training in toddlers, which have resulted in improving specific aspects of navigational skills without general effects on navigation *tout court* (Boccia et al., 2017).

Turning from the evidence showing alteration of the cerebral network (i.e., the insula, the lingual gyrus, the inferior and superior frontal gyri in the right hemisphere) involved in learning spatial sequences in the environmental space in the presence of PTSD as a consequence of natural disasters (e.g., Piccardi et al., 2016a), the present results suggest that the increase in the capability to learn topographic sequences in EPs might be modulated by specific brain circuits that have not been altered, thus playing a key role in positive coping mechanisms. It may be possible that a restructured cognitive map that requires substitutions, insertions and deep changes may produce an observable effect at the behavioral and, possibly, at the neural level. Nevertheless, this issue needs further investigations.

Despite the novelty of these results, it should be recognized that there was no baseline evaluation of topographic memory in the EPs. However, it is unlikely that participants of the exposed group were accidentally recruited with higher topographic memory skills, especially as the scores on the Semmes Test and FSCS were not different between the two groups. However, a longitudinal design that involves testing participants during the aftermath and in the short, medium and long term after trauma exposure is needed to confirm the present data. Moreover, future studies should conduct longitudinal investigations to assess clinical populations with different severities of psychological distress to better understand whether topographic memory should be considered a protective factor to prevent mild cognitive impairments and/or psychological trauma-induced sequelae. The collection of salivary cortisol samples to measure psychological stress may also be useful in future studies as the absence of these physiological measures in the present study do not allow conclusions to be drawn with respect to the mechanisms underlying adaptational plasticity. To fully demonstrate our interpretation, future studies should use a spatial updating task to more directly explore the idea that environmental changes foster the acquisition and storing of new spatial knowledge, thus resulting in an improved cognitive map of the environment. We limited our investigation to TL, which is undoubtedly the process underlying the formation of the mental map, but this is just a small part of the numerous processes underlying spatial navigation.

Another limitation of the present study was the absence of measures taken before the earthquake. There is no evidence regarding the EPs’ mental maps before the disaster; therefore, it was not possible to make comparisons with their new mental maps. However, by observing Figure 1, it is reasonable to assume that important environmental changes occurred and that these changes required individuals to update previous mental maps. The NEP group learned the new town after they moved to L’Aquila following the earthquake. The absence of differences within the NEP group between visuospatial, verbal and topographic memory seems to suggest that this kind of topographic practice did not produce an enhancement of topographic memory. Their answers to the FSCS showed that they were confident in moving around L’Aquila city without experiencing topographic disorientation episodes. Therefore, the two groups cannot be considered equivalent with respect to the degree of familiarity with the city, a factor that has importance in human navigation proficiency (e.g., Nori and Piccardi, 2011; Lopez et al., 2018).

In conclusion, these results may shed light on positive, long-term changes that occur in environmental mental representation mechanisms after exposure to a natural disaster. To our knowledge, this aspect has not been previously investigated and deserves further study to better understand cognitive map formation under post-traumatic stress in the absence of clinical disorders.

## Author Contributions

LP, MP, MB, AMG and SDA conceived and designed the experiment. AB, FB and PC collected data. MP and MB analyzed data and all authors contributed to the writing of the article.

## Conflict of Interest Statement

The authors declare that the research was conducted in the absence of any commercial or financial relationships that could be construed as a potential conflict of interest.
